# First Description of *Mergibacter septicus* Isolated from a Common Tern (*Sterna hirundo*) in Germany

**DOI:** 10.3390/pathogens12091096

**Published:** 2023-08-28

**Authors:** Mostafa Y. Abdel-Glil, Silke Braune, Sandra Bouwhuis, Lisa D. Sprague

**Affiliations:** 1 Institut für Bakterielle Infektionen und Zoonosen (IBIZ), Friedrich-Loeffler-Institut, 07743 Jena, Germany; mostafa.abdelglil@fli.de; 2 Department of Pathology, Faculty of Veterinary Medicine, Zagazig University, Zagazig 44511, Egypt; 3 Niedersächsisches Landesamt für Verbraucherschutz und Lebensmittelsicherheit (LAVES), Lebensmittel- und Veterinärinstitut Braunschweig/Hannover, 30173 Hannover, Germany; silke.braune@laves.niedersachsen.de; 4 Institut für Vogelforschung, 26386 Wilhelmshaven, Germany; sandra.bouwhuis@ifv-vogelwarte.de

**Keywords:** *Mergibacter septicus*, common tern, bird, Germany, genome, taxonomy, *Pasteurella*

## Abstract

*Mergibacter septicus* (*M. septicus*), previously known as Bisgaard Taxon 40, is a recently described species within the *Pasteurellaceae* family. In this study, we present a *M. septicus* strain isolated from a common tern (*Sterna hirundo*) chick that died just after fledging from the Banter See in Wilhelmshaven, Germany. The recovered *M. septicus* strain underwent microbiological phenotypic characterization, followed by whole genome sequencing on Illumina and Nanopore platforms. Phenotypically, *M. septicus* 19Y0039 demonstrated resistance to colistin, cephalexin, clindamycin, oxacillin, and penicillin G. The genome analysis revealed a circular 1.8 Mbp chromosome without any extrachromosomal elements, containing 1690 coding DNA sequences. The majority of these coding genes were associated with translation, ribosomal structure and biogenesis, followed by RNA processing and modification, and transcription. Genetic analyses revealed that the German *M. septicus* strain 19Y0039 is related to the American strain *M. septicus* A25201^T^. Through BLAST alignment, twelve putative virulence genes previously identified in the *M. septicus* type strain A25201^T^ were also found in the German strain. Additionally, 84 putative virulence genes distributed across nine categories, including immune modulation, effector delivery system, nutrition/metabolic factors, regulation, stress survival, adherence, biofilm, exotoxin, and motility, were also identified.

## 1. Introduction

Migratory birds can carry and disperse emerging (zoonotic) pathogens and establish new endemic foci at great distances from the origin of infection [1,2]. Among these emerging pathogens are West Nile virus (WNV), avian influenza (AI), Newcastle disease virus (ND), and tick-borne encephalitis virus (TBE). Further emerging bacterial, fungal, and protozoan pathogens include, among others, several enteropathogens, such as *Salmonella* spp. and *Campylobacter* (*C.*) *jejuni* as well as the causative agents of avian botulism (*Clostridium* (*C.*) *botulinum*), avian cholera (*Pasteurella* (*P.*) *multocida*), aspergillosis *(Aspergillus* spp.), and avian malaria (*Plasmodium* spp.). These pathogens pose a particular threat to endangered species. Monitoring of dead birds has become an increasingly important factor within the context of nature conservation and thus the preservation of these animals [1,2].

Common terns (*Sterna hirundo*) are relatively long-lived, piscivorous, migratory seabirds in the order of *Charadriiformes* and family *Laridae* and members of the genus *Sterna*. Terns inhabit coastal areas and inland waters and can be found almost worldwide. They frequently form large nesting colonies and play a key role as an indicator species reflecting the state of the marine environment and the overall condition of coastal habitats. Common tern populations face numerous mortality challenges including infectious diseases [3,4], environmental stressors, chemical compounds, and habitat changes that affect nesting success and overall health [5]. In Germany, common terns are listed as “severely endangered” in the Red List of Endangered Species [6]. A large breeding colony of common terns with approximately 650 breeding pairs is located at the Banter See in Wilhelmshaven, Germany. This colony has been under constant surveillance of the Institute of Avian Research in Wilhelmshaven since 1992 [7].

The *Pasteurellaceae* family comprises a large collection of Gram-negative bacteria commonly found in numerous mammal and bird species; they can cause severe disease in humans and animals. *Haemophilus influenzae*, for example, is associated with various respiratory tract infections, such as pneumonia, bronchitis, and sinusitis, and may lead to invasive disease, e.g., meningitis in young children. *Pasteurella multocida* is pathogenic to numerous animal species, including cats, dogs, and birds, whereas *Mannheimia haemolytica* affects cattle and other ruminants. In recent years, the taxonomy of the *Pasteurellaceae* family has undergone major revisions and advancements, leading to the discovery of novel genera and species [8]. 

*Mergibacter septicus* is a bacterium of the *Pasteurellaceae* family, previously known as Bisgaard Taxon 40. Although little is known regarding its ecological habitat, transmission, and pathogenicity, it has been associated with multi-species mortality events in seabirds, including common and sandwich terns, as well as Rhinoceros Auklets (*Cerorhinca monocerata*) [9,10]. Affected birds presented with pneumonia, neurological signs, and septicaemia [9,10,11]. To date, reports on *M. septicus* have been sporadic and largely geographically confined to the USA (Florida; Washington) despite affecting migratory avian species. To our knowledge, this is the first report on the isolation of *M. septicus* in Germany, possibly Europe. 

## 2. Materials and Methods

### 2.1. Clinical Case Description, Post-Mortem Examinations, and Histologic Examinations 

Between 30 June and 18 July 2019, 22 adult common terns breeding at the Banter See colony as well as chicks of various ages were found dead. One adult and 8 chicks were sent to the Lower Saxony State Office for Consumer Protection and Food Safety for investigation. Exemplary post-mortem examinations were performed on two juveniles, one of which was in poor body condition, whereas the other was emaciated. Significant gross findings included alveolar edema, alveolar emphysema, and congestion of the lungs and liver. Histologic examination of lung sections showed vascular congestion in both birds and alveolar histiocytosis as well as multifocal basophilic bacterial structures in one. Routine bacterial cultures were performed on selected tissues (intestine, liver, and lung) on Columbia blood agar plates containing 5% sheep blood incubated at 37 °C. Among others, Pasteurella-like organisms were isolated from the lung tissue of one of the juveniles and sent to the Institute of Bacterial Infections and Zoonoses at the Friedrich-Loeffler-Institut in Jena for further analyses. 

### 2.2. Cultivation and Identification of M. septicus 19Y0039 

The isolate was cultivated on Columbia blood agar containing 5% sheep blood and incubated at 37 °C. Matrix-assisted laser desorption ionisation time-of-flight (MALDI-TOF) mass spectrometry analysis (UltrafleXtreme, Bruker Daltonics Inc., Billerica, MA, USA) was used according to the manufacturer’s instructions for presumptive genus and species identification. Biochemical profiles and carbon source utilisation were assessed by means of Analytical Profile Index (API) test strips 20E, API50 CH, and APIZYM (all from Biomerieux GmbH, Nürtingen, Germany) according to the manufacturer’s instructions.

### 2.3. Antimicrobial Susceptibility Testing of M. septicus 19Y0039

The minimum inhibitory concentration (MIC) was determined by broth microdilution using the automated MICRONAUT-S system (Micronaut-S MDR-MRGN Screening and Micronaut-S small animal, MERLIN Diagnostics GmbH, Bornheim-Hersel, Germany) according to the manufacturer’s instructions. The results were evaluated automatically with MICRONAUT-S software as susceptible, intermediate, or resistant (Supplementary Table S1). The MIC values were interpreted according to the Clinical and Laboratory Standards Institute (CLSI) break point guidelines available for *Pasteurella* spp. The reference strains *Escherichia (E.) coli* ATCC 25922, *E. coli* NCTC 13846, and *Pseudomonas* (*P.*) *aeruginosa* ATCC 27853 were used for quality control. Colistin MIC values were additionally determined by ETEST^®^ (BioMerieux GmbH) on Mueller Hinton agar containing 5% sheep blood according to the manufacturer’s instructions.

### 2.4. Genome Sequencing of M. septicus Isolate 19Y0039

Genomic DNA was extracted using the QIAGEN^®^ Genomic-tip 100/G kit and the Genomic DNA Buffer Set (QIAGEN GmbH, Hilden, Germany) according to the manufacturer’s instructions. The DNA purity was assessed with a Colibri spectrophotometer (Thermo Fisher Scientific, Schwerte, Germany) and was quantified using a Qubit 3 Fluorometer with the Qubit^TM^ double-stranded DNA (dsDNA) high-sensitivity (HS) Assay Kit (Invitrogen^TM^, Schwerte, Germany). The paired-end genome sequencing library was generated with the Nextera XT DNA Library Preparation Kit (Illumina Inc., San Diego, CA, USA) followed by whole genome sequencing using an Illumina MiSeq instrument to generate reads 300 bp in length according to the manufacturer’s instructions.

Additionally, genome sequencing was performed on MinION (Oxford Nanopore Technology, Oxford, UK) using the ONT 1D Ligation Sequencing Kit (SQK-LSK109) according to the manufacturer’s instructions. The final DNA library containing 225 ng DNA was loaded onto an R9.4.1 flow cell (FLO-MIN106) with 1442 available pores for sequencing.

### 2.5. Genomic Characterisation of M. septicus

The genome assembly of *M. septicus* 19Y0039 was performed by using ONT long reads followed by polishing with the Illumina data. Processing of the ONT data generated with the MinION sequencer was performed as follows: base calling of the raw FAST5 data was performed using the ONT Guppy base calling software (v6.1.5+446c35524) with the config file for super accurate base calling model (dna_r9.4.1_450bps_sup.cfg). The base called ONT FASTQ reads were filtered and trimmed using the porechop (v0.2.4; https://github.com/rrwick/Porechop; Accessed on 22 August 2023) and filtlong (v0.2.1; https://github.com/rrwick/Filtlong; Accessed on 22 August 2023) tools, respectively. Nanoq (v0.9.0) was used to report summary statistics of the obtained FASTQ nanopore data [12]. Next, long-read-only assembly was performed with Flye (v2.9) [13] using the following parameters: “--nano-hq and flye_polishing_iterartions = 2”. Assembly polishing with the long reads was performed with four rounds of Racon (v1.5.0) [14] in combination with minimap2 (v2.24-r1122) and, finally, one round of polishing with Medaka (v1.6.1.). Polypolish (v0.5.0) in combination with bwa (v0.7.17) was then used to correct the ONT-based assembly with Illumina reads using standard settings [15,16]. Genome statistics encompassing genome length and total number of assembled contigs was obtained with SeqKit (v2.3.0) [17]. 

The taxonomic identification of *M. septicus* 19Y0039 was performed by initially extracting the 16S rRNA sequences using barrnap (v0.93; available at https://github.com/tseemann/barrnap; Accessed on 22 August 2023). Additionally, 16S rRNA gene sequences from further members of the *Pasteurellaceae* family (*n* = 71) were included for comparison. All extracted 16S rRNA genes were aligned with mafft (v7.307) [18] followed by phylogenetic analysis by constructing a maximum likelihood tree with the FastTree program [19]. Subsequently, Average Nucleotide Identity (ANI) was calculated using pyani (v0.2.3) in the Mummer module (ANIm) [20]. PhyloPhlAn (v0.43) was also used for phylogenetic taxonomic analysis [21]. PhyloPhlAn utilized an optimized reference set of marker genes and employed a phylogenetic approach to accurately assign taxonomy to an investigated sample set [21].

For genome annotation, Bakta software (v1.5.1) with Bakta database (v4.0) was used in default settings [22]. Core genome-based phylogeny was performed using Parsnp (v1.2) within the Harvest suite with default settings [23]. 

### 2.6. Antibiotic Resistance and Virulence-Related Genes

The presence of known antibiotic resistance genes was investigated in public databases using ABRicate (v1.0.1; https://github.com/tseemann/abricate; Accessed on 22 August 2023). Abricate uses the BLAST+ algorithm and searches for AMR genes in NCBI, ResFinder [24], CARD [25], and ARG-ANNOT [26]. All databases were updated on 10 January 2023. In addition, the AMRfinderplus tool (v3.10.42) with database (v2022-10-11.2) was used with default settings to search for resistance genes [27]. 

Putative virulence genes previously reported by De Luca et al. [11] including a capsular gene, genes encoding for outer membrane proteins (*omp*A, *omp*H), a superoxide dismutase (*sod*A), a cytolethal distending toxin (*cdt*), as well as genes involved in lipooligosaccharide (LOS) synthesis (*gal*U, *gal*E, *lpx*A, *lpx*C, and *kds*A) and iron metabolism (*fur* and *exb*D) were searched for with BLASTN v 2.2.9 in *M. septicus* 19Y0039. The Fasta sequences of the putative virulence genes were kindly provided by Dr. De Luca and colleagues upon request. 

Finally, a BLAST analysis of protein sequences (BLASTp) was performed against the full set of genes present in the virulence factor database for a more extensive search of virulence-related genes [28,29]. Thresholds were set for e-value < 1 × 10^−20^. The results of the BLASTP analysis were filtered to limit BLAST hits to a query sequence identity above 50% and the query coverage above 80%. Annotations of the identified genes were then extracted from the Bakta annotation results of the strain [22].

## 3. Results and Discussion

### 3.1. Phenotypic Characteristics of M. septicus 19Y0039

*M. septicus* 19Y0039 grows aerobically on blood agar at 28 °C, 37 °C, and 42 °C with ß-haemolysis. Colonies are small, round, and shiny with a whitish-cream colony morphology, and Gram-negative rods can be seen under the microscope, in agreement with the findings of De Luca et al. [11]. No growth was observed on MacConkey agar. *M. septicus* 19Y0039 is catalase- and oxidase-positive, but urease and indole tests are negative. Acid is formed from D-glucose, D-mannose, and D-sorbitol. Enzymatic activity was observed for C4 esterase, leucine arylamidase, acid phosphatase, and naphtol-AS-BI-phosphohydrolase at 37 °C and 42 °C. No bacterial identification was obtained by MALDI-TOF analysis.

### 3.2. Antimicrobial Susceptibility Testing of M. septicus 19Y0039

The antimicrobial resistance phenotype of *M. septicus* 19Y0039 was evaluated after incubation at 37 °C and 42 °C, respectively (Supplementary Table S1). In contrast to the findings of De Luca et al. (2021) [11] with regard to antibiotic susceptibility of the isolates used to describe the newly proposed genus and species *Mergibacter septicus*, the automated MICRONAUT-S system found *M. septicus* 19Y0039 to be resistant to colistin, cephalexin, clindamycin, oxacillin, and penicillin G. ETEST^®^ (BioMerieux GmbH) determined the Colistin MIC value to be at 2 µg/mL.

Migratory birds are not only sentinels and reservoirs for antibiotic resistance but also play a pivotal role in the dissemination of resistant bacteria [30,31]. Although no genetic determinants for resistance were found in *M. septicus* 19Y0039, it displayed resistance to several antibiotics in vitro. Resistance to oxacillin, cephalexin, erythromycin, and clindamycin has been described previously for *Pasteurella* spp. [32,33,34]. The impact of the observed colistin resistance remains elusive, as no cut-off values for colistin in *Pasteurella* spp. exist to date. However, not further determined colistin resistant bacterial species have been isolated from numerous migrating avian species, such as Arctic tern, white stork, lesser black-backed gull, and European herring gull [31,35,36,37].

### 3.3. Genome Description of M. septicus 19Y0039

**Figure 1:**
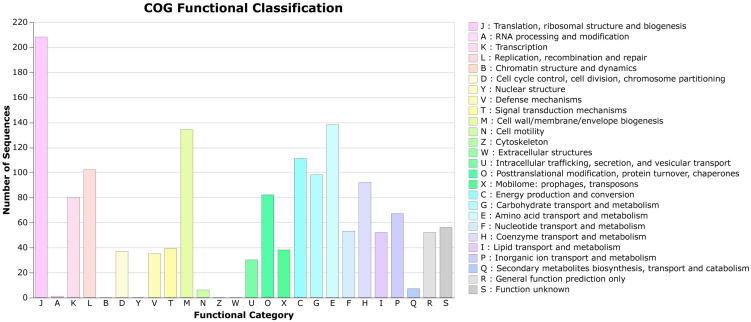
COG functional classifications of the *Mergibacter septicus* genes.

Table 1 shows the sequencing metrics of *M. septicus* 19Y0039 generated with Illumina and Oxford Nanopore sequencing. Genome assembly resulted in one circular chromosome without extrachromosomal elements. The total length of the chromosome was 1,889,691. The GC content was 35.9%. Genome annotation resulted in a total of 59 tRNAs, 19 rRNAs, seven ncRNAs, 11 ncRNA regions, and one CRISPR array. The coding density of the genome was estimated to be 90% with a total of 1689 coding DNA sequences (CDSs), including 102 genes encoding for hypothetical proteins. Three pseudogenes were predicted in the genome. The genomic features of the sequenced strain agree with those recently described for *Mergibacter septicus* gen. and sp. nov. in terms of genome size and content. A total of 89.82% (1518/1690) of the sequences were classified into COG functional categories (Figure 1). The majority of genes belonged to the category translation, ribosomal structure, and biogenesis, followed by RNA processing and modification, then transcription. In total, 82 genes belonged to the “defense mechanisms” category.

### 3.4. Taxonomic Classification of M. septicus 19Y0039 from Germany

**Figure 2:**
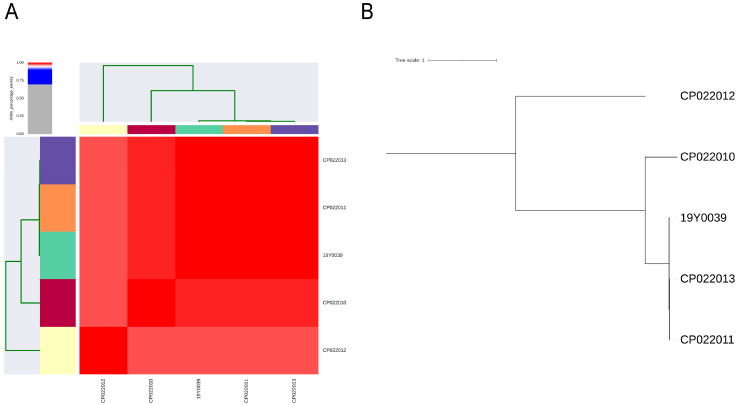
Genetic relatedness of German *Mergibacter septicus* strain 19Y0039 with American strains A25201^T^ (CP022010), CP022011, CP022012, and CP022013. (**A**) Percentage values of average nucleotide identity between all strains. (**B**) Core genome based phylogenetic analysis of all strains obtained with Parsnp.

The 16S rRNA gene analysis of the German strain revealed near complete conformity with the American *Mergibacter septicus* spec. nov. A25201^T^ (CP022010), CP022011, and CP022013 strains, but 12 SNPs were detected between the German strain and the American strain CP022012. For all other members belonging to the *Pasteurellaceae* group, a minimum of 76 SNPs were detected in the 16S rRNA gene. The pairwise genomic average nucleotide identity was estimated to be between 98.1% and 99.9% compared to the *M. septicus* genomes (CP022010, CP022011, CP022013, and CP022012; Figure 2A), demonstrating that all genomes belonged to the same species. The results of phylophlan confirmed the species assignment of the German strain to the recently described genus *Mergibacter* gen. nov., with one amino acid difference found between the German isolate and the American isolates CP022013 and CP022011, 18 amino acid differences to the type strain CP022010, and 43 to the isolate CP022012. This calculation was based on a concatenated alignment of 35,489 amino acid positions of up to 400 universally conserved bacterial proteins as reported with the phylophlan software. 

Despite genetic analyses revealing that the German *M. septicus* strain 19Y0039 is related to the American strain *M. septicus* A25201^T^, the origin of infection in the birds investigated in the present study could not be determined. The majority of German Common Terns breed in the Wadden Sea and along the Baltic coast; however, some birds can also be found in inland colonies along rivers and lakes. The majority of birds breeding in the Wadden Sea as well as those breeding in the western and southern German inland spend the winter along the western African coasts [38], whereas those from the Baltic coast and the eastern German are inland along the southern African coasts [39]. American Common Terns, on the other hand, spend the winter in South America or along the coast of Central America [40]. How *M. septicus* spreads between continents and which putative vectors are involved are currently unknown and need further research.

### 3.5. Virulence Genes in M. septicus

BLAST alignment resulted in the identification of twelve putative virulence genes previously discovered in the *M. septicus* type strain A25201^T^. The sequences of the virulence genes were highly conserved between the strains (>98% sequence similarity and 100% sequence coverage compared to the type of strain). The genes involved in lipooligosaccharide synthesis and toxin production displayed more non-synonymous SNPs in comparison to the genes involved in iron metabolism and outer membrane protein synthesis (Supplementary Table S2). 

Next, we assessed the extent of virulence gene homologues in the *M. septicus* strain 19Y0039 by retrieving all virulence genes in the VFDB. BLAST analysis of the protein sequences against the VFDB predicted 84 putative virulence genes distributed in nine categories, i.e., immune modulation, effector delivery system, nutrition/metabolic factor, regulation, stress survival, adherence, biofilm, exotoxin, and motility (Supplementary Table S3). Among these were 47 genes with high amino acid sequence identity to virulence markers of the genus *Haemophilus*. These included genes involved in iron metabolism, such as heme biosynthesis genes (*hemA*, *hemB*, *hemC*, *hemD*, *hemE*, *hemG*, *hemH*, *hemL*, *hemN*, *hemN*, and *hemY*) and *hitABC* genes. The latter three genes were arranged in a gene cluster, which may indicate an operon structure. Similarly, the *sitABCD* gene cluster (involving four genes) was found to resemble the *sitABCD* of the avian pathogenic *Escherichia coli* (APEC) strain χ7122, which mediated iron and manganese transport and resistance to hydrogen peroxide [41]. In addition, *M. septicus* strain 19Y0039 was found to possess type IV pili genes (*pilB*, *ppdD*, *vfr*, and *comE*/*pilQ*) that played a role in cell adhesion, colonisation, and motility of Gram-negative bacteria. Moreover, a set of 23 lipo-oligosaccharide related genes have been identified that show high similarity to the lipooligosaccharide (LOS) genes of different *Haemophilus* species. LOS is an important virulence element in *Haemophilus* spp., responsible for adherence of the bacterial strain to the host substrate and resistance to complement and other host antimicrobial factors. Four more gene homologues were discovered that may have been involved in exopolysaccharide production. The genes have been detected previously in *Haemophilus somnus* and are thought to contribute to colonisation during early natural infection [42]. Despite the presence of putative virulence genes, the real pathogenic potential of *M. septicus* cannot be currently determined. Further studies from recognised clinical cases or from experimental infections are necessary to determine its pathogenic potential.

## 4. Conclusions

Migratory birds are exposed to a plethora of pathogens that can cause severe disease and ultimately death. The detection of these pathogens can be difficult due to the logistical challenges encountered when monitoring and investigating mortality events in the wild. Additionally, pathogen isolation can be severely hampered by overgrowth of, or growth suppression through, colonising bacteria. Despite genetic similarities to the American strain *M. septicus* A25201^T^, the origin of the German *M. septicus* 19Y0039 strain remains elusive. The in-depth analysis of *M. septicus* 19Y0039, however, has demonstrated the importance of combining phenotypic and genetic analyses with regard to antibiotic resistance, as it was not possible to link the observed resistance in vitro with currently known genetic determinants in silico. Other causes leading to antibiotic resistance such as mutations in, e.g., metabolic genes, are still difficult to identify, as they are time consuming to induce, and extensive databases with conclusive information on metabolic networks are missing. Nonetheless, this study was able to determine further putative virulence genes corroborating the observed pathogenicity of *M. septicus* in seabirds. Further studies are required to determine the routes of transmission and possible zoonotic potential. 

## Figures and Tables

**Table 1:** Genome characteristics of strain *M. septicus* 19Y0039.

Isolation source	Host of isolation	Common tern (*Sterna hirundo*)
	Year of isolation	2019
	Geography	Germany
Illumina sequencing	Total number of nucleotides	510,359,003
	Total number of reads	2,111,510
	Mean read length (bp)	241 (forward), 242 (reverse)
	Q30 bases	439,668,245 (86.1%)
	Mean sequencing depth	270×
Nanopore sequencing	Total number of nucleotides	2,499,984,591
	Total number of reads	499,799
	N50 read length (bp)	8602
	Mean read length (bp)	5001
	Mean sequencing depth	1268 ± 121×
Assembly and annotation	Total number of contigs	1
	Chromosome size (Mb)	1,889,691/(1,887,770) *
	G + C content (%)	35.91%/(36.4%) *
	Total number of coding sequences	1690/(1693) *
	Total number of rRNA operons	6
	Total number of tRNA	58/(58) *

* Values in brackets denote the reported metrics for *M. septicus* A25201^T^ (accession# CP022010).

## Data Availability

The sequence data of the isolate 19Y0039 was submitted to the National Center for Biotechnology Information (NCBI) under the bio-project accession number PRJNA1009712.

## Supplementary Materials

The following supporting information can be downloaded at: https://www.mdpi.com/article/10.3390/pathogens12091096/s1. Table S1: Results of antimicrobial resistance testing in *M. septicus* 19Y0039. Table S2: Sequence conservation of the virulence factors in *M. septicus* 19Y0039. Table S3: Potential virulence factors identified in the *M. septicus* strain.

## References

[1] Reed K.D., Meece J.K., Henkel J.S., Shukla S.K. (2003). Birds, migration and emerging zoonoses: West nile virus, lyme disease, influenza A and enteropathogens. Clin. Med. Res..

[2] Fuller T., Bensch S., Müller I., Novembre J., Pérez-Tris J., Ricklefs R.E., Smith T.B., Waldenström J. (2012). The ecology of emerging infectious diseases in migratory birds: An assessment of the role of climate change and priorities for future research. Ecohealth.

[3] Pohlmann A., Stejskal O., King J., Bouwhuis S., Packmor F., Ballstaedt E., Hälterlein B., Hennig V., Stacker L., Graaf A. (2023). Mass mortality among colony-breeding seabirds in the German Wadden Sea in 2022 due to distinct genotypes of HPAIV H5N1 clade 2.3.4.4b. J. Gen. Virol..

[4] Strauch E., Jäckel C., Hammerl J.A., Hennig V., Roschanski N., Dammann I. (2020). Draft Genome Sequences of *Vibrio cholerae* Non-O1, Non-O139 Isolates from Common Tern Chicks *Sterna hirundo* following a Mass Mortality Event. Microbiol. Resour. Announc..

[5] Soerensen A.L., Faxneld S. The Use of Common Tern, Eurasian Oystercatcher, and Great Cormorant as Indicator Species for Contaminant Monitoring. https://www.diva-portal.org/smash/get/diva2:1422813/FULLTEXT01.pdf.

[6] Ryslavy T., Bauer H.-G., Gerlach B., Hüppop O., Stahmer J., Südbeck P., Sudfeldt C. (2020). Rote Liste der Brutvögel Deutschlands. 6 Fassung. Berichte zum Vogelschutz.

[7] Moiron M., Charmantier A., Bouwhuis S. (2022). The quantitative genetics of fitness in a wild seabird. Evolution.

[8] Michael G.B., Bossé J.T., Schwarz S. (2018). Antimicrobial resistance in *Pasteurellaceae* of veterinary origin. Antimicrob. Resist. Bact. Livest. Companion Anim..

[9] Knowles S., Bodenstein B.L., Berlowski-Zier B.M., Thomas S.M., Pearson S.F., Lorch J.M. (2019). Detection of Bisgaard Taxon 40 in Rhinoceros Auklets (*Cerorhinca monocerata*) with Pneumonia and Septicemia from a Mortality Event in Washington, USA. J. Wildl. Dis..

[10] Niedringhaus K.D., Shender L.A., DiNuovo A., Flewelling L.J., Maboni G., Sanchez S., Deitschel P.J., Fitzgerald J., Nemeth N.M. (2021). Mortality in Common (*Sterna hirundo*) and Sandwich (*Thalasseus sandvicensis*) Terns Associated with Bisgaard Taxon 40 Infection on Marco Island, Florida, USA. J. Comp. Pathol..

[11] De Luca E., Álvarez-Narváez S., Maboni G., Baptista R.P., Nemeth N.M., Niedringhaus K.D., Ladner J.T., Lorch J.M., Koroleva G., Lovett S. (2021). Comparative Genomics Analyses Support the Reclassification of Bisgaard Taxon 40 as *Mergibacter* gen. nov., With *Mergibacter septicus* sp. nov. as Type Species: Novel Insights into the Phylogeny and Virulence Factors of a *Pasteurellaceae* Family Member Associated with Mortality Events in Seabirds. Front. Microbiol..

[12] Steinig E., Coin L. (2022). Nanoq: Ultra-fast quality control for nanopore reads. J. Open Source Softw..

[13] Kolmogorov M., Yuan J., Lin Y., Pevzner P.A. (2019). Assembly of long, error-prone reads using repeat graphs. Nat. Biotechnol..

[14] Vaser R., Sović I., Nagarajan N., Šikić M. (2017). Fast and accurate de novo genome assembly from long uncorrected reads. Genome Res..

[15] Li H., Durbin R. (2009). Fast and accurate short read alignment with Burrows-Wheeler transform. Bioinformatics.

[16] Wick R.R., Holt K.E. (2022). Polypolish: Short-read polishing of long-read bacterial genome assemblies. PLoS Comput. Biol..

[17] Shen W., Le S., Li Y., Hu F. (2016). SeqKit: A Cross-Platform and Ultrafast Toolkit for FASTA/Q File Manipulation. PLoS ONE.

[18] Katoh K., Standley D.M. (2013). MAFFT multiple sequence alignment software version 7: Improvements in performance and usability. Mol. Biol. Evol..

[19] Price M.N., Dehal P.S., Arkin A.P. (2010). FastTree 2—Approximately Maximum-Likelihood Trees for Large Alignments. PLoS ONE.

[20] Pritchard L., Glover R.H., Humphris S., Elphinstone J.G., Toth I.K. (2016). Genomics and taxonomy in diagnostics for food security: Soft-rotting enterobacterial plant pathogens. Anal. Methods.

[21] Segata N., Börnigen D., Morgan X.C., Huttenhower C. (2013). PhyloPhlAn is a new method for improved phylogenetic and taxonomic placement of microbes. Nat. Commun..

[22] Schwengers O., Jelonek L., Dieckmann M.A., Beyvers S., Blom J., Goesmann A. (2021). Bakta: Rapid and standardized annotation of bacterial genomes via alignment-free sequence identification. Microb. Genom..

[23] Treangen T.J., Ondov B.D., Koren S., Phillippy A.M. (2014). The Harvest suite for rapid core-genome alignment and visualization of thousands of intraspecific microbial genomes. Genome Biol..

[24] Bortolaia V., Kaas R.S., Ruppe E., Roberts M.C., Schwarz S., Cattoir V., Philippon A., Allesoe R.L., Rebelo A.R., Florensa A.F. (2020). ResFinder 4.0 for predictions of phenotypes from genotypes. J. Antimicrob. Chemother..

[25] Jia B., Raphenya A.R., Alcock B., Waglechner N., Guo P., Tsang K.K., Lago B.A., Dave B.M., Pereira S., Sharma A.N. (2017). CARD 2017: Expansion and model-centric curation of the comprehensive antibiotic resistance database. Nucleic Acids Res..

[26] Gupta S.K., Padmanabhan B.R., Diene S.M., Lopez-Rojas R., Kempf M., Landraud L., Rolain J.M. (2014). ARG-ANNOT, a new bioinformatic tool to discover antibiotic resistance genes in bacterial genomes. Antimicrob. Agents Chemother..

[27] Feldgarden M., Brover V., Gonzalez-Escalona N., Frye J.G., Haendiges J., Haft D.H., Hoffmann M., Pettengill J.B., Prasad A.B., Tillman G.E. (2021). AMRFinderPlus and the Reference Gene Catalog facilitate examination of the genomic links among antimicrobial resistance, stress response, and virulence. Sci. Rep..

[28] Liu B., Zheng D., Jin Q., Chen L., Yang J. (2019). VFDB 2019: A comparative pathogenomic platform with an interactive web interface. Nucleic Acids Res..

[29] Altschul S.F., Madden T.L., Schaffer A.A., Zhang J., Zhang Z., Miller W., Lipman D.J. (1997). Gapped BLAST and PSI-BLAST: A new generation of protein database search programs. Nucleic Acids Res..

[30] Bonnedahl J., Järhult J.D. (2014). Antibiotic resistance in wild birds. Upsala J. Med. Sci..

[31] Prakash E.A., Hromádková T., Jabir T., Vipindas P., Krishnan K., Hatha A.M., Briedis M. (2022). Dissemination of multidrug resistant bacteria to the polar environment-Role of the longest migratory bird Arctic tern (*Sterna paradisaea*). Sci. Total Environ..

[32] Goldstein E., Citron D., Richwald G. (1988). Lack of in vitro efficacy of oral forms of certain cephalosporins, erythromycin, and oxacillin against Pasteurella multocida. Antimicrob. Agents Chemother..

[33] Citron D.M., Warren Y.A., Fernandez H.T., Goldstein M.A., Tyrrell K.L., Goldstein E.J. (2005). Broth microdilution and disk diffusion tests for susceptibility testing of Pasteurella species isolated from human clinical specimens. J. Clin. Microbiol..

[34] Eid H.M., Algammal A.M., Elfeil W.K., Youssef F.M., Harb S.M., Abd-Allah E.M. (2019). Prevalence, molecular typing, and antimicrobial resistance of bacterial pathogens isolated from ducks. Vet. World.

[35] Loucif L., Chelaghma W., Cherak Z., Bendjama E., Beroual F., Rolain J.M. (2022). Detection of NDM-5 and MCR-1 antibiotic resistance encoding genes in *Enterobacterales* in long-distance migratory bird species *Ciconia ciconia*, Algeria. Sci. Total Environ..

[36] Jarma D., Sánchez M.I., Green A.J., Peralta-Sánchez J.M., Hortas F., Sánchez-Melsió A., Borrego C.M. (2021). Faecal microbiota and antibiotic resistance genes in migratory waterbirds with contrasting habitat use. Sci. Total Environ..

[37] Kenzaka T. (2020). Genome sequence of colistin-resistant *Escherichia coli* CLR8, isolated from the feces of *Larus argentatus*. Microbiol. Resour. Announc..

[38] Kürten N., Schmaljohann H., Bichet C., Haest B., Vedder O., González-Solís J., Bouwhuis S. (2022). High individual repeatability of the migratory behaviour of a long-distance migratory seabird. Mov. Ecol..

[39] Piro S., Schmitz Ornés A. (2022). Revealing different migration strategies in a Baltic Common Tern (*Sterna hirundo*) population with light-level geolocators. J. Ornithol..

[40] Bracey A., Lisovski S., Moore D., McKellar A., Craig E., Matteson S., Strand F., Costa J., Pekarik C., Curtis P. (2018). Migratory routes and wintering locations of declining inland North American Common Terns. Auk.

[41] Sabri M., Léveillé S., Dozois C.M. (2006). A SitABCD homologue from an avian pathogenic *Escherichia coli* strain mediates transport of iron and manganese and resistance to hydrogen peroxide. Microbiology.

[42] Corbeil L.B. (2007). *Histophilus somni* host–parasite relationships. Anim. Health Res. Rev..

